# Nasal cytology and histology in CRSwNP: Two sides of the same coin

**DOI:** 10.3389/fmed.2023.1143351

**Published:** 2023-03-09

**Authors:** Matteo Gelardi, Rossana Giancaspro, Michele Cassano, Domenico Ribatti

**Affiliations:** ^1^Department of Clinical and Experimental Medicine, Unit of Otolaryngology, University Hospital of Foggia, Foggia, Italy; ^2^Department of Translational Biomedicine and Neurosciences, University of Bari Medical School, Bari, Italy

**Keywords:** CRSwNP, nasal cytology, histology, mast cells, eosinophils

## Abstract

Knowledge of chronic rhinosinusitis with nasal polyps (CRSwNP) has increased rapidly over the past decade. However, the study of the histological features of nasal polyps has not gone hand in hand with the study of the inflammatory mechanisms underlying CRSwNP. Indeed, precisely because they are benign neoformations, nasal polyps have not attracted the attention of pathologists over the years. Nasal cytology has shown that CRSwNP, generally defined as a Type-2 disease, is characterized not only by eosinophilic but also mast cell inflammation and, in particular, the most severe forms of CRSwNP are precisely characterized by a mixed eosinophilic-mast cell inflammation. Interestingly, mast cells cannot be visualized by histology due to limitations in staining and magnification, and therefore are not commonly described in histological reports of nasal polyps. However, immunohistochemistry can highlight these latter cells and specifically this technique has recently demonstrated that mast cells are located in the lamina propria of almost all types of polyps and in the epithelial level of the most severe forms. Unfortunately, the latter technique is not commonly carried out in clinical practice by virtue of the high cost and time burden. On the other hand, nasal cytology is an easy-to-apply and economic diagnostic tool, commonly practiced in rhinological setting, which can effectively fill the gap between histology and immunohistochemistry, allowing to non-invasively establish the endotype of nasal polyps and to highlight all cytotypes, including mast cells, that cannot be visualized by the other two techniques. The recent demonstration of the close correlation between mast cell intraepithelial infiltrate and CRSwNP severity paves the way for new therapeutic possibilities aimed at reducing not only eosinophilic infiltration but also mast cell infiltration.

## Introduction

Knowledge about Chronic Rhinosinusitis with Nasal Polyps (CRSwNP) has increased rapidly in the last decade, in parallel with the advent of biological agents (1). As a matter of fact, promising results of biological treatment on asthmatic patients with comorbid CRSwNP have progressively highlighted that CRSwNP, in the upper airway, and asthma, in the lower airway, often share underlying pathophysiology, namely type 2 inflammation, according to the “unified airway disease theory” (2). Therefore, type 2-targeting biologics such as anti-IgE, anti-IL4Rα, anti-IL4/IL-13, anti-IL5, and anti-IL5Rα, which have entered the market for selected phenotypes/endotypes of asthma patients, are nowadays available or will be available for CRSwNP patients (3).

Unfortunately, the study of the histological characteristics of nasal polyps has not gone hand in hand with the study of the inflammatory mechanisms underlying CRSwNP. Indeed, precisely because they are benign neoformations, over the years nasal polyps have not attracted the attention of pathologists and pathological evaluations have not been adapted to the current knowledge about the etiopathogenetic inflammatory mechanisms underlying the disease. This is the reason why histological reports of nasal polyps often end up being mere “copy and paste,” mainly useful for excluding differential diagnoses but not for characterizing polyps, despite the importance for an accurate diagnosis (4).

While histological reports are limited to describing nasal polyps as benign neoplasms composed of an edematous, fibrotic or lax myxoid stroma covered by respiratory epithelium with a predominantly eosinophilic inflammatory pattern, nasal cytology has shown that not only eosinophils but also other cytotypes infiltrate nasal polyps, influencing their severity and tendency to recurrence (5). In particular, the evaluation of nasal immunophlogosis, together with that of comorbidities, allows to establish a Clinical-Cytological Grading (CCG), which determines the severity of the CRSwNP and the Prognostic Index of Recurrence (PIR) (6).

## Nasal polyps from a histological point of view

Hippocrates firstly described nasal polyps in his book Diseases II, written in Greek. The term polyp comes from the Greek π*ωλυπ*oζ (pôlupos), meaning octopus, or polypus in Latin. He believed that the etiology of nasal polyps was represented by a modification of phlegm and that the treatment consisted of removal of the feet of the polyp by pulling on a sponge ball, cauterization, a snare, or possibly using a knife *via* an external approach (7). However, unlike intestinal polyps, nasal polyps do not have a fibrovascular axis, as they are protrusions of the sino-nasal mucosa that lines a fibro-myxoid stroma, without a vascular axis. Therefore, the mere excision of the nasal polyp at its base of implantation does not prevent its recurrence (8, 9).

By virtue of progressive histological knowledge, nasal polyps were classified into four different histological patterns: edematous, fibroinflammatory, with pronounced hyperplasia of seromucous glands and with atypical stroma. The most common type was represented by edematous so-called “allergic” polyps, characterized by edema, goblet cell hyperplasia of the epithelium, thickening of the basement membrane, and of numerous leukocytes, predominantly eosinophils. The second histological type was the fibroinflammatory polyp, characterized by chronic inflammation and metaplastic changes of the overlying epithelium. Rare variants were represented by polyps with, respectively pronounced hyperplasia of seromucous glands and with atypical stroma (10). Over time, however, this classification was abandoned.

Today, nasal polyps are defined as benign, non-neoplastic inflammatory outgrowth of sino-nasal mucosa characterized by edematous stroma infiltrated by mixed inflammatory cells. In particular, the edematous, fibrotic or loosely myxoid stroma is covered by respiratory epithelium, which is infiltrated by mixed inflammatory cells, including lymphocytes, plasma cells, eosinophils, neutrophils and mast cells (MCs). The surface epithelium can show ulceration or squamous metaplasia or rarely osseous metaplasia. Stroma cells can be bizarre, large, and pleomorphic. Submucosal glands are decreased or absent. Concurrent fungal infection may be seen (11).

Typically, biopsy specimens of nasal polyps are fixed in 10% buffered formalin and subjected to normal processing by paraffin embedding and hematoxylin-eosin (HE) staining. This staining, together with the 40X magnification used by pathologists, does not allow to visualize all the cells that infiltrate the stroma and the epithelium, especially the MCs.

## Nasal cytology

**Figure 1:**
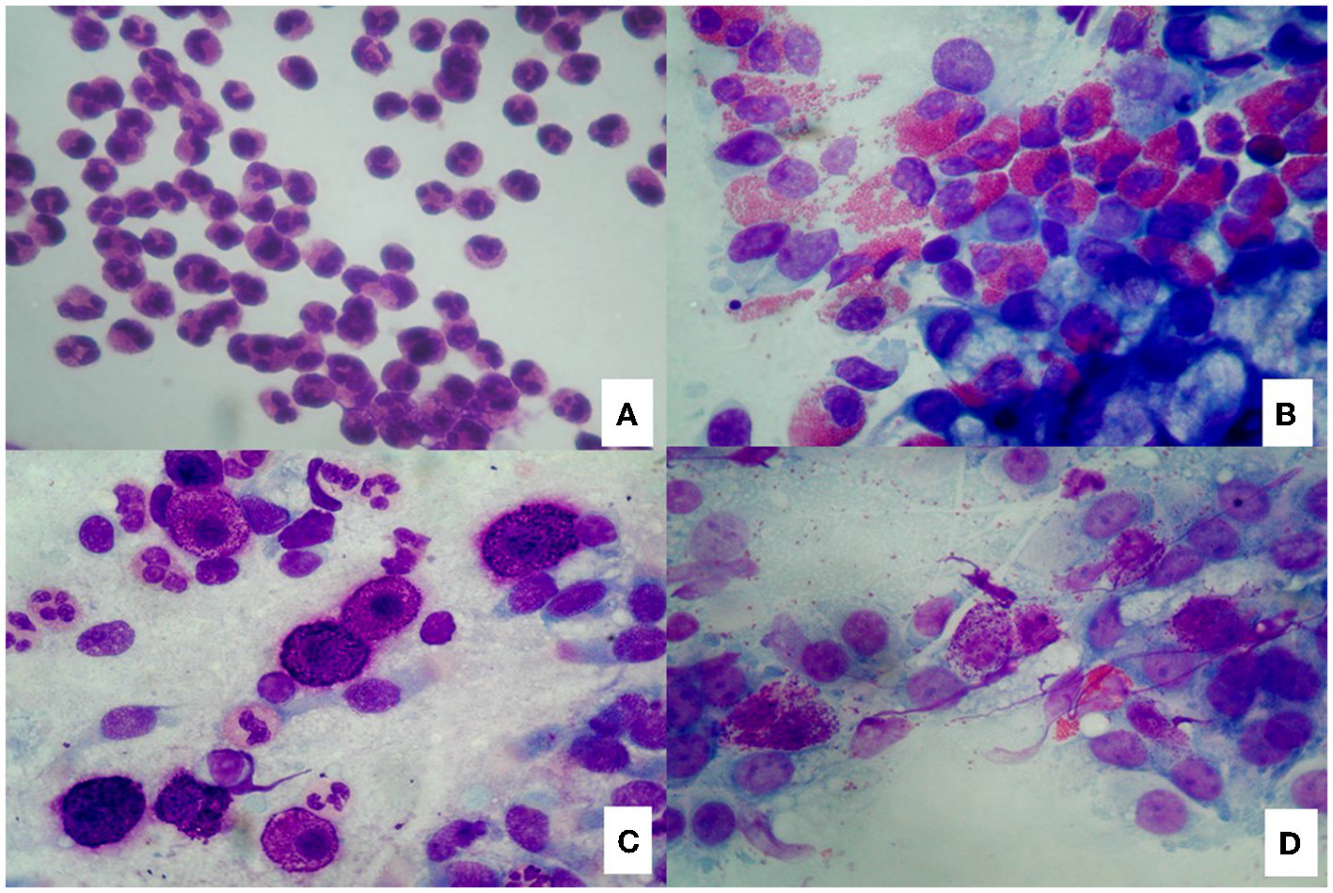
Nasal cytology. **(A)** Neutrophils; **(B)** Eosinophils; **(C)** Mast cells; **(D)** Eosinophils and mast cells. MGG staining. Magnification 1000x.

Nasal cytology (NC) is a non-invasive diagnostic tool that has become an integral part of the diagnostic process of sino-nasal disorders to evaluate the degree of nasal immunophlogosis. According to validated criteria, cytological samples are obtained under anterior rhinoscopy, from the middle part of the inferior turbinate, immediately smeared on a glass side and air-dried. Then, samples are stained with May-Grunwald-Giemsa (MGG) and read with a 1,000 x objective with oil immersion. A minimum of fifty fields is considered necessary to identify a sufficient number of cells (12). Four cytologic endotypes can be identified: neutrophilic, eosinophilic, mast-cell and mixed cellularity (eosinophil and mast-cells) (Figures 1A–D). According to the different cytotypes and comorbidities, a CCG can be established to assess the severity of CRSwNP and the PIR. In particular, a score is assigned to each comorbidity (ASA sensitivity corresponds to one, asthma to two, allergy to three, ASA sensitivity combined to asthma to three) as well to each inflammatory cell pattern as well (neutrophilic corresponds to one, mast cell to two, eosinophilic to three, mixed eosinophilic-mast cell to four) and the total CCG score is given by the sum of the scores attributed to comorbidities and inflammatory pattern.

In turn, the CCG is closely related to the PIR, which reflects the possibility of relapse. Indeed, depending on the score, the patient may have a low (CCG ≤ 3), medium (4 < CCG < 7), or high risk (CCG ≥ 7) of recurrence. Therefore, each phenotype and endotype contributes differently to determining the severity of the disease.

Since it is a bloodless procedure, it should be empathized that NC evaluates the predominant inflammatory cells infiltrating the epithelium, and that the eosinophil-mast cell endotype is considered the most difficult to treat, with the greatest risk of recurrence. The strong correlation between eosinophil-mast cell infiltration and severity of CRSwNP has been further confirmed by recent studies conducted with immunofluorescence and confocal laser microscopy on nasal polyps biopsies (13). In particular, Galectin-10, which is traditionally considered a marker of eosinophilic inflammation, has been shown to colocalize with both eosinophils and MCs, being directly related to disease severity.

The noteworthy aspect of NC is that this technique also allows to characterize from a cellular point of view any patient affected by CRSwNP, even without undergoing invasive procedures such as surgery and biopsy. As a matter of fact, not all patients suffering from CRSwNP undergo surgical treatment, indeed the first treatment is mostly represented by intranasal corticosteroids. Only about 18% of patients undergo functional endoscopic sinus surgery (FESS) within the first year of being diagnosed with CRSwNP. In this context, NC is particularly important to define the severity of the diseases of unoperated patients even before surgery, without performing any invasive procedure.

## The link between histology and nasal cytology

The histological examination of HE-stained specimens of nasal polyps does not allow to reveal the presence of MCs due to the limitations of both the staining and the magnification. Nevertheless, thanks to NC, MCs are nowadays known not only to infiltrate nasal polyps but, above all, to play a crucial role in the pathogenesis of CRSwNP, orchestrating eosinophilic inflammation and causing the most severe forms, usually refractory to traditional treatments (14).

Immunohistochemistry could be a link between histology and nasal cytology, being able to reveal the presence of inflammatory cells, in particular MCs, through the use of specific monoclonal antibodies against Tryptase (mouse monoclonal primary antibody, clone G3) and anti-CD117 (15, 16). As a matter of fact, recent studies under publication, conducted with immunohistochemistry on biopsy samples of nasal polyps, have shown that MCs infiltrate the *lamina propria* of almost all nasal polyps and the epithelial layer of most difficult to treat forms.

In particular, the intraepithelial localization of MCs is directly related to the severity of CRSwNP, according to the CCG, and a cut-off of intraepithelial MCs could be defined to identify the most severe endotypes of CRSwNP. NC has already proven to be a valid tool for identifying the inflammatory cells infiltrating the epithelial level. On the contrary, by definition NC cannot reveal the presence of inflammatory cells in the *lamina propria*, being limited to the evaluation of the epithelial level. The correlation found between cytological examination and immunohistochemical histological examination in the detection of intraepithelial MCs not only underlines the reliability of NC in examining nasal immunophlogosis but pave the way to new considerations.

**Figure 2:**
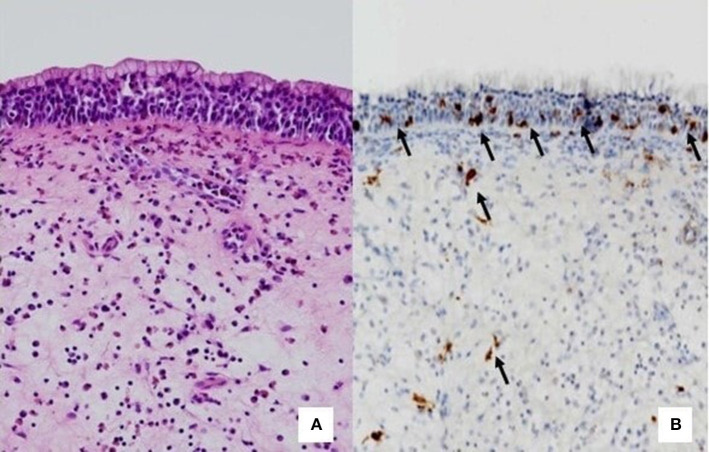
Comparison between histology and immunohistochemistry. **(A)** HE staining that do not highlight mast cells. **(B)** Immunohistochemistry. The arrows indicate CD117+ mast cells, located at the intraepithelial level or in the lamina propria.

Until recently, eosinophils were considered the pivotal cells of CRSwNP, followed by neutrophils. Over time, the dualism between eosinophils and neutrophils has been re-evaluated and finally MCs have been shown to be directly involved in the pathogenesis of CRSwNP, orchestrating type-2 cytokines and chemokines (17, 18). Once the crucial role of MCs is understood, it has been demonstrated that even the different localization of MCs, at the intraepithelial level or in the lamina propria, can differently influence the severity of the disease (Figures 2A, B). However, since MCs and eosinophils appear to influence each other, it is unclear whether MCs are recruited in the epithelial level of nasal polyps by massive eosinophilic inflammation or whether other factors influence mast cell recruitment.

The main limit of immunohistochemistry is represented by the economic and time burden. This technique, as well as immunofluorescence and flow cytometry, allows for the phenotyping of nasal polyps with extreme precision. However, precisely the high costs and the high expenditure of time limit the use of these methods in clinical practice and reserve them mainly for research purposes.

Therefore, the combined cytological-histological evaluation can represent the right compromise: histology, in fact, is fundamental to exclude differential diagnoses; cytology, on the other hand, allows to evaluate the inflammatory infiltrate of the nasal polyps at the epithelial level and, consequently, to establish the severity of the pathology. Given the close correlation found between immunohistochemistry and NC, immunohistochemistry would not seem indispensable in the diagnostic/therapeutic process, being a much more invasive and costly method than NC.

## Consequences of the cyto-histological evaluation of CRSwNP: The emerging role of MCs in CRSwNP and potential therapeutic implications

In the last twenty years, it has been clearly demonstrated that MCs play a crucial role in diseases other than allergy, including cardiovascular and autoimmune diseases, and various cancer types (19). Only recently, the complementary evaluation of nasal polyps from both a cytological and histological point of view has allowed to bring to light new aspects, among which an abundant mast cell infiltrate in the lamina propria and in the epithelium of the nasal polyps, directly correlated to the severity of the disease.

MCs are strategically localized at the junction point of the host and external environment in the gastro-intestinal tract, skin, and respiratory epithelium, where they are localized at the interface between inner organs and outer microenvironment, impeding the interaction with different pathogens (20). In these different contexts, MCs play an anti-microbial, anti-viral, and anti-parasite effects and contribute to the activation of other inflammatory cells in the infected tissues (21). These cells are recruited to the anatomic site of inflammatory process, where they release numerous mediators and cytokines, which contribute to the recruitment of other inflammatory cells, to the clearance of debris and pathogens, to the induction of angiogenesis and to the activation of fibroblasts to synthesize extracellular matrix components. Moreover, MCs able to release different anti- and pro-inflammatory agents, modulating their role from protective to pro-inflammatory cells in different pathological conditions. As a matter of fact, different mast cell phenotypes develop in different tissues and in different locations of the same tissue. In particular, on the basis of tissue micro localization and protease expression, two major human MCs subtypes have been identifies: sub epithelial MCs (MC_TC_), coexpressing tryptase and chymase in conjunction with cathepsin G and carboxypeptidase A3 (CPA3), and mucosal epithelial MCs (MC_T_), expressing only tryptase. Type 2 CRSwNP and asthma are characterized by the expansion of intraepithelial MC_T_ expressing CPA3 and MC_TC_ infiltrating the airway smooth muscle and subepithelial glandular tissue (22). Interestingly, MCs recruit circulating eosinophils through direct secretion of eosinophil-attracting chemokines, such as eotaxins, and indirectly *via* secretion of histamine, inducing eotaxin secretion by endothelial cells. Together MCs and eosinophils are considered key effectors not only in allergy but also in Type 2 diseases and can be considered an “Effector Unit,” communicating with each other in a bidirectional manner.

This crosstalk is mediated by physical cell–cell contact through cell surface receptors/ligands and by released mediators, including specific granular factors, chemokines, cytokines, and their respective ligands. Therefore, through complex cellular processes, MCs can recruit eosinophils, increase vessel permeability and influence the induction, amplitude, and function of the adaptive immune response (23). This explains what nasal cytology had already demonstrated: the eosinophilic-mast cell forms of CRSwNP are the most severe and difficult to treat, as they are subtended by a dense network of cytokines and chemokines which contribute to the perpetuation of inflammation and thus to the generation of polyps nasals (24).

## Concluding remarks

A complementary cytological and histological evaluation is fundamental in the diagnostic-therapeutic pathway of CRSwNP. While histology allows for the exclusion of differential diagnoses, this technique fails to identify the endotype of nasal polyps. In contrast, immunohistochemistry allows the detection of all cytotypes of the nasal infiltrate and, consequently, the identification of the different endotypes. Nevertheless, the latter procedure is much more invasive and expensive than NC, which still allows to evaluate all the cytotypes infiltrating the nasal mucosa, but in an economical and non-invasive way. Furthermore, the NC allows to characterize from a cellular point of view also nasal polyps of patients who do not undergo surgery, allowing to establish their severity.

Therefore, we believe that histology and cytology but not immunohistochemistry should be performed in all patients affected by CRSwNP also in order to choose patient-tailored therapies.

In this context, the adoption of medical strategies targeting not only eosinophils but also mast cells appears essential to effectively treat CRSwNP. Biological therapies available today specifically focus on eosinophilic response, implementing a strategy of blocking the eosinophils. However, in the light of current knowledge, it is also essential to act on MCs. Reducing MCs number is a therapeutic approach in mastocytosis and other diseases in which MCs number is increased. Number of MCs may be reduced by the targeted induction of apoptosis or by blocking their recruitment, migration and differentiation. The therapeutic approach used in diseases in which mast cell number is increased includes tyrosine kinase inhibitors (midostaurin, nilotinib, imatinib and dasatinib) to target the cKIT receptor action and mast cell tryptase inhibitors (gabexatemesylate, nafamostat mesylate, and tranilast) (25). Moreover, mast cell stabilizing drugs inhibit the release of mediators are used to prevent allergic reactions to common allergens. More recently, mast cell stabilizing agents, such as cromolyn sodium, have been used in different pre-clinical models of solid tumors (26).

Further studies are needed to better understand the mechanisms underlying the interaction between eosinophils and MCs as well as to clearly define the different endotypes of CRSwNP, which are not limited to eosinophilic or neutrophilic forms. Furthermore, defining therapeutic strategies tailored to the patient, which target all the cytotypes and molecules involved in the pathogenesis of CRSwNP, based on the precise stratification of patients according to their phenotype and endotype, represents the future goal, in the context of precision medicine.

## Data availability statement

The raw data supporting the conclusions of this article will be made available by the authors, without undue reservation.

## Author contributions

MG and DR conceived the study and reviewed the final manuscript. RG wrote the manuscript. MC reviewed the manuscript. All authors contributed to the article and approved the submitted version.
